# Genomic and transcriptomic analyses reveal selection of genes for puberty in Bama Xiang pigs

**DOI:** 10.24272/j.issn.2095-8137.2018.068

**Published:** 2018-06-19

**Authors:** Yang Yang, Adeniyi C. Adeola, Hai-Bing Xie, Ya-Ping Zhang

**Affiliations:** 1 State Key Laboratory of Genetic Resources and Evolution, Yunnan Laboratory of Molecular Biology of Domestic Animals, Kunming Institute of Zoology, Chinese Academy of Sciences, Kunming Yunnan 650204, China; 2 Kunming College of Life Science, University of Chinese Academy of Sciences, Kunming Yunnan 650204, China

**Keywords:** Puberty, Bama Xiang pig, Pituitary, Differentially expressed genes

## Abstract

The Bama Xiang pig (BMX) is a famous early-maturing Chinese indigenous breed with a two-end black coat. To uncover the genetic basis of the BMX phenotype, we conducted comparative genomic analyses between BMX and East Asian wild boars and Laiwu pigs, respectively. Genes under positive selection were enriched in pathways associated with gonadal hormone and melanin synthesis, consistent with the phenotypic changes observed during development in BMX pigs. We also performed differentially expressed gene analysis based on RNA-seq data from pituitary tissues of BMX and Large White pigs. The *CTTNBP2NL*, *FRS2*, *KANK4*, and *KATNAL1* genes were under selection and exhibited expressional changes in the pituitary tissue, which may affect BMX pig puberty. Our study demonstrated the positive selection of early maturity in the development of BMX pigs and advances our knowledge on the role of regulatory elements in puberty evolution in pigs.

## INTRODUCTION

Pigs are important protein food source and globally distributed animal domesticated from Eurasian wild boars 9 000 years ago (Larson et al., 2005). During their long domestication history, pigs have exhibited abundant phenotypic variation in coat color, reproduction, and growth. This phenotypic diversity provides valuable animal models for genetic studies (Ciobanu et al., 2001; Rubin et al., 2012).

The Bama Xiang pig (BMX) from Guangxi Zhuang Autonomous Region is a well-known variety noted for its early maturity and two-end black pigmentation. The female BMX pig matures at about three months, in sharp contrast to the five months observed in most other East Asian domestic pigs (China National Commission of Animal Genetic Resources, 2011). Thus, the BMX pig provides an important model to study early maturity and pigmentation evolution during pig domestication.

In this study, we performed genomic screening of signatures of positive selection in the BMX pig in comparison with those of the East Asian wild boar (EAW) and Laiwu (LWU) pigs. The LWU pig has a normal maturity process (at six months) and black coat color (China National Commission of Animal Genetic Resources, 2011), but shares the same domestication origin as that of the BMX pig from East Asian wild boars (Wu et al., 2007). In combination with transcriptomic analysis, we identified genetic variations that were putatively associated with the maturity and pigment phenotype in the BMX pig. The primary goal of this study was to explore the (1) genomic evolutionary pattern shaped by development of the BMX pig, and (2) genetic variations associated with selection for early maturity and coat color in the BMX pig.

## MATERIALS AND METHODS

### Sample collection and resequencing of genomic DNA

We downloaded the genome resequencing data of six BMX, six LWU, and 13 EAW pigs from the NCBI database (Supplementary Table S1), as reported from earlier studies (Ai et al., 2015; Groenenet al., 2012; Li et al., 2013; Rubin et al., 2012). Sequence reads were filtered by removing adaptors and low-quality bases using Trimmomatic (Bolger et al., 2014). Genomic reads were aligned to the *Sus scrofa* 10.2 genome (Groenenet al., 2012) with the BWA program (Li & Durbin, 2010). Alignment was performed with a maximum of six mismatches allowed for each 100-bp fragment.

### Single nucleotide polymorphism (SNP) calling and annotation

SNP calling was performed with the DNA sequencing data of the 25 pig genomes using HaplotypeCaller in the GATK toolkit (McKenna et al., 2010). Only polymorphic SNPs were included in this analysis. VariantFiltration and SelectVariants were used to filter SNPs by quality score (QUAL <20) and depth of coverage (DP <10). SNPs were annotated with the GTF file downloaded from the NCBI website (https://www.ncbi.nlm.nih.gov/). For annotation, SNPs were classified by their locations in protein-coding (synonymous or non-synonymous), intronic, intergenic, and untranslated region (UTR) sequences.

### Selective sweep detection

Population fixation index (*F*_ST_) (Weir & Cockerham, 1984), cross population extended haplotype homozygosity (XPEHH) (Sabeti et al., 2007), and nucleotide diversity (π) (Tajima, 1983) were used to search for signals of selection in the BMX pig genome. We analyzed selective sweeps in 10-kb non-overlapping sliding windows. Windows containing <5 SNPs were removed from the analysis.

The *F*_ST_ values between BMX and other pigs (LWU and EAW) were calculated using VCFtools (Danecek et al., 2011).

Haplotypes were inferred using Beagle software (Browning & Browning, 2007). Comparing the BMX pigs with LWU and EAW pigs, XPEHH was calculated to examine haplotypes that showed low levels of linkage decay in the BMX populations (Coop et al., 2009; Pickrell et al., 2009; Pritchard et al., 2010).

The nucleotide diversities of BMX (π_BMX_), EAW (π_EAW_), and LWU (π_LWU_) pigs were then calculated separately using VCFtools (Danecek et al., 2011). To identify regions that showed low nucleotide diversity in the BMX pigs compared to the LWU and EAW pigs, we calculated π_EAW_ divided by π_BMX_ and π_LWU_ divided by π_BMX_, respectively.

Genomic windows showing high *F*_ST_, low nucleotide diversity, and high XPEHH values were identified as having signatures of selection in BMX pigs. Candidate selective sweeps were chosen from genomic windows identified simultaneously by all three statistics as having values above the top 10% threshold in their empirical distributions.

### RNA sample collection, sequencing, and identification of differentially expressed genes

Pituitary tissues were collected from three BMX pigs and three Large White pigs for RNA sequencing (RNA-seq). These pigs were females at 85 days after birth. The RNA-seq libraries with an inserted size of 250 bp were prepared using the Illumina standard RNA-seq library preparation pipeline and sequenced on the Illumina Hiseq 2000 platform, with 150-bp paired-end reads generated. In total, the transcriptomes of six pituitaries (GSA number: CRA000876) were used for comparative analysis. The RNA-seq clean reads were mapped onto the *Sus scrofa* 10.2 genome using Tophat2 (Kim et al., 2013). A new merged GTF annotation file was generated using Cufflinks and Cuffmerge (Trapnell et al., 2010).

Differentially expressed genes (DEGs) were identified by DESeq2 (Love et al., 2014). As DESeq2 required a raw read count table as input, we obtained the raw read count using HTseq (Anders et al., 2014). An FDR of <0.05 was used as the cutoff for DEGs. The ClueGO plugin in Cytoscape (Mlecnik et al., 2018) was used for gene ontology (GO) enrichment analysis. GO terms with a *P*-value of <0.05 were defined as enriched.

## RESULTS

### Selective signatures in BMX pig genomes

**Figure 1:**
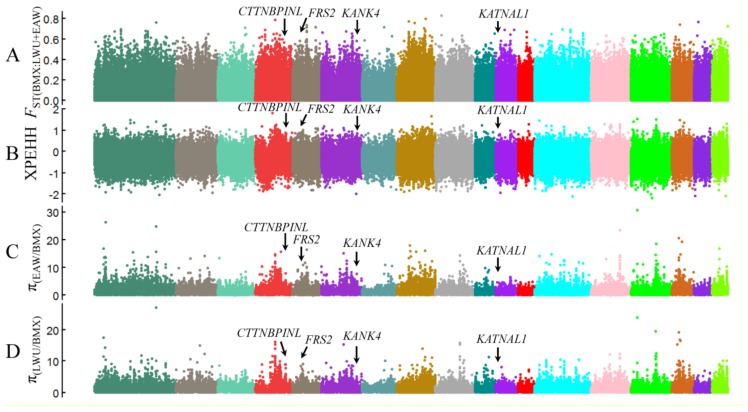
Whole genome selective signatures of BMX pigs

The whole genomes of six BMX, 13 EAW, and six LWU pigs were used to identify genomic variants underlying the development of BMX pigs. Genomic reads were quality filtered and mapped to the pig reference genome (*Sus scrofa* 10.2) (see Materials and Methods). A total of 43.5 million SNPs were identified in the 25 individuals. To identify signatures of selection in BMX pig genomes, we analyzed the *F*_ST_ (Weir & Cockerham, 1984), π (Tajima, 1983), and XPEHH (Sabeti et al., 2007) metrics along chromosomes in comparison to those of EAW and LWU pigs (Figure 1). Selective signatures were screened in 10-kb non-overlapping sliding windows and adjacent selective windows were combined. As recent artificial selection produces long stretches of haplotypes in a population (Amaral et al., 2008), a clustering of selective windows with intervals of less than nine windows was chosen as an indicator of a putatively selective sweep. Sweep regions greater than 30 kb in length were included in the following analyses. This procedure resulted in the identification of 257 genes within 339 putative selective sweeps in the BMX pig genomes (Supplementary Table S2).

X-axis shows the positions of the windows along the 1–18 autosome chromosomes. Different chromosomes are displayed in different colors. Y-axis shows the *F*_ST_ (A), XPEHH (B), π_EAW/BMX_ (C), and π_LWU/BMX_ (D) values. *F*_ST_ and XPEHH values were calculated between BMX and (LWU+EAW) pigs. π_EAW/BMX_ and π_LWU/BMX_ were obtained from π_EAW_ and π_LWU_ divided by π_BMX_, respectively.

Functional enrichment analysis of the gene set revealed significant overrepresentation of 59 terms (Table 1). Interestingly, several biological processes related to sexual maturity were involved, including “pituitary gland development” (*BMP4*, *FGF10*, and *TBX19*), “endocrine system development” (*BMP4*, *CRH*, *FGF10*, *NEUROD1*, and *TBX19*), and “negative regulation of reproductive process” (*BMP4*, *CSN2*, and *ZP4*). The pituitary is the most important endocrine gland involved in growth and reproduction regulation. In addition, five genes (*FGF10*, *FGF2*, *MITF*, *PDGFC*, and *PDGFD*) were involved in “Melanoma”, which might be associated with the two-end black pigmentation observed in BMX pigs, which differs from most Chinese domestic pigs with black coats (China National Commission of Animal Genetic Resources, 2011).

**Table 1:** Functional enrichment terms of positively selected genes in BMX pigs.

GO ID	GO term	*P-*value^*^
GO:0004080	Neuroactive ligand-receptor interaction	24.0E-15
GO:0060449	Bud elongation involved in lung branching	100.0E-6
GO:0032740	Positive regulation of interleukin-17 production	140.0E-6
GO:0060393	Regulation of pathway-restricted SMAD protein phosphorylation	230.0E-6
GO:0060389	Pathway-restricted SMAD protein phosphorylation	250.0E-6
GO:0032946	Positive regulation of mononuclear cell proliferation	310.0E-6
GO:0050671	Positive regulation of lymphocyte proliferation	310.0E-6
GO:0070665	Positive regulation of leukocyte proliferation	370.0E-6
GO:0048636	Positive regulation of muscle organ development	400.0E-6
GO:0045844	Positive regulation of striated muscle tissue development	400.0E-6
GO:1901863	positive regulation of muscle tissue development	450.0E-6
GO:0005412	Arrhythmogenic right ventricular cardiomyopathy (ARVC)	490.0E-6
GO:0005218	Melanoma	560.0E-6
GO:0045740	Positive regulation of DNA replication	600.0E-6
GO:0009798	Axis specification	600.0E-6
GO:0090090	Negative regulation of canonical Wnt signaling pathway	600.0E-6
GO:0032660	Regulation of interleukin-17 production	660.0E-6
GO:0051155	Positive regulation of striated muscle cell differentiation	660.0E-6
GO:0032620	Interleukin-17 production	780.0E-6
GO:0042506	Tyrosine phosphorylation of Stat5 protein	780.0E-6
GO:0051145	Smooth muscle cell differentiation	800.0E-6
GO:0061037	Negative regulation of cartilage development	920.0E-6
GO:0048546	Digestive tract morphogenesis	950.0E-6
GO:0005410	Hypertrophic cardiomyopathy (HCM)	990.0E-6
GO:0051149	Positive regulation of muscle cell differentiation	1.2E-3
GO:0035270	Endocrine system development	1.3E-3
GO:0055025	Positive regulation of cardiac muscle tissue development	1.3E-3
GO:0042310	Vasoconstriction	1.5E-3
GO:0060602	Branch elongation of an epithelium	1.5E-3
GO:0001709	Cell fate determination	2.0E-3
GO:0021536	Diencephalon development	1.8E-3
GO:0021983	Pituitary gland development	2.2E-3
GO:0042307	Positive regulation of protein import into nucleus	2.3E-3
GO:0000561	Glycerolipid metabolism	2.4E-3
GO:0004730	Long-term depression	2.6E-3
GO:0003338	Metanephros morphogenesis	2.4E-3
GO:0030199	Collagen fibril organization	2.4E-3
GO:0060441	Epithelial tube branching involved in lung morphogenesis	3.0E-3
GO:0030219	Megakaryocyte differentiation	3.3E-3
GO:0042383	Sarcolemma	3.3E-3
GO:0009799	Specification of symmetry	3.3E-3
GO:0051153	Regulation of striated muscle cell differentiation	3.3E-3
GO:0010092	Specification of organ identity	3.3E-3
GO:1900182	Positive regulation of protein localization to nucleus	3.7E-3
GO:0005033	Nicotine addiction	4.0E-3
GO:0004140	Regulation of autophagy	5.9E-3
GO:2000242	Negative regulation of reproductive process	5.9E-3
GO:0070664	Negative regulation of leukocyte proliferation	5.1E-3
GO:0003401	Axis elongation	5.1E-3
GO:0009948	Anterior/posterior axis specification	5.5E-3
GO:0055024	Regulation of cardiac muscle tissue development	5.1E-3
GO:0004940	Type I diabetes mellitus	7.4E-3
GO:0048259	Regulation of receptor-mediated endocytosis	6.9E-3
GO:0007193	Adenylate cyclase-inhibiting G-protein coupled receptor signaling pathway	11.0E-3
GO:0009880	Embryonic pattern specification	11.0E-3
GO:0005160	Transforming growth factor beta receptor binding	9.5E-3
GO:0031623	Receptor internalization	11.0E-3
GO:0048286	Lung alveolus development	9.5E-3
GO:0010862	Positive regulation of pathway-restricted SMAD protein phosphorylation	10.0E-3

^*^: *P-*value obtained from ClueGO plugin in Cytoscape using default paraments. Only terms with *P*<0.05 are listed.

Although many missense mutations are associated with the domestication of pigs (Fang et al., 2009; Li et al., 2010; Ren et al., 2011; Rubin et al., 2012), whether regulatory variants play a role and their effects on the domestication processes compared to coding variants remain unknown. To address this issue, we compared the evolutionary patterns for SNPs identified from the selective regions in the BMX pig genomes. A total of 830 949 SNPs were identified from 339 sweeps. Among these, 3 668 SNPs were located in coding sequences and 827 542 SNPs were located in noncoding sequences. We first focused on protein coding region variations located in the 339 sweeps. We examined highly differentiated missense mutations and analyzed their presence in DNA sequences encoding conserved protein sequences. We concentrated on missense SNPs with *F*_ST_ values up to the chromosomal top 0.1% threshold and compared BMX pigs to LWU and EAW pigs, respectively. We identified a dataset consisting of 11 missense SNPs from 10 genes (Supplementary Table S3). Alignment of the 10 protein sequences showed that the flanking sequences at the residues encoded by the 11 SNPs displayed low level cross-species conservation, implying weak functional constraints on these protein-coding variants. This indicated that regulatory elements rather than missense variants may have greater effects on BMX pig development.

### Comparative transcriptomic analysis of pituitary tissues

We conducted transcriptomic profiling to examine gene expression changes due to selection in the BMX genomes as regulatory variants exhibited a profound role during BMX pig development. We chose pituitary tissue as the pituitary gland secretes reproduction-related hormones involved in maturity regulation. In our experimental design, we collected pituitary tissues from three BMX and three Large White pigs at 85 days after birth. At this stage, BMX pigs are close to maturity, whereas Large White pigs do not reach maturity until eight months of age (China National Commission of Animal Genetic Resources, 2011). The transcriptomes from the pituitary tissues were sequenced, from which we obtained 35 million mapped reads, on average, for each individual. We compared the gene expression levels in the pituitary and found 1 600 DEGs between BMX and Large White pigs. Although Large White pigs were domesticated from European wild boars (Larson et al., 2005;Rubin et al., 2012), the DEGs between BMX and Large White pigs provided valuable information on expressional changes in BMX pigs if they also exhibited selective signatures in the BMX genome. The DEGs were enriched in the following biological processes: “Estrogen signaling pathway” (*ADCY6*, *ADCY8*, *CREB3L1*, *CREB5*, *FOS*, *GABBR2*, *GNAS*, *GRM1*, *KCNJ9*, *MMP9*, *PIK3CG*, *PIK3R5*, and *PRKACA*), “Ovarian steroidogenesis” (*ADCY6*, *ADCY8*, *ALOX5*, *GNAS*, *LDLR*, *LHCGR*, and PRKACA), “Oocyte meiosis” ( *ADCY6*, *ADCY8*, *CAMK2B*, *CAMK2D*, *CCNE1*, *CDC20*, *CPEB2*, *ESPL1*, *MAD2L2*, *MOS*, *PRKACA*, and *YWHAZ*), “Developmental maturation” ( *C12H17orf47*, *CDC20*, *EDNRB*, *FAM101B*, *LTF*, *PLP1*, *PRKACA*, *RBPJ*, *TAL1*, *WASH1*, and *WNT10B*), and “Progesterone-mediated oocyte maturation” ( *ADCY6*, *ADCY8*, *CPEB2*, *MAD2L2*, *MOS*, *PIK3CG*, *PIK3R5*, and *PRKACA*) (Supplementary Table S4). These pathways all participate in sexual maturity.

**Figure 2:**
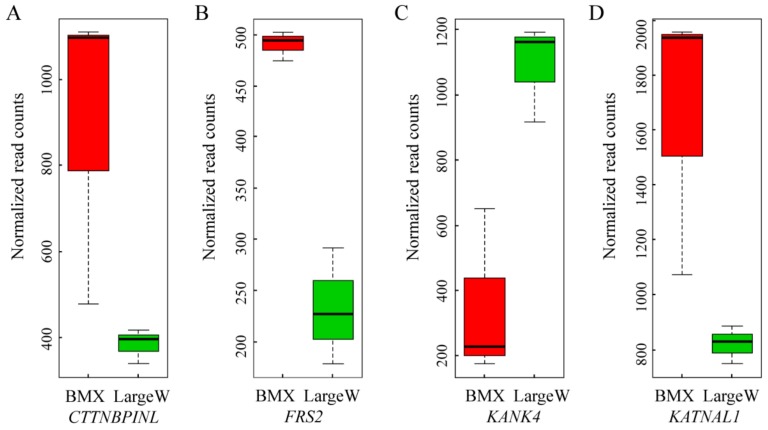
Normalized read counts of significantly differentially expressed genes *CTTNBP2NL* (A), *FRS2* (B), *KANK4* (C), and *KATNAL1* (D) in the pituitary

As both artificially selected genes and DEGs showed functional enrichment in maturity regulation, it is possible that differential gene expression was favored by the artificial selection of variants from some sweep loci. We therefore identified artificially selected genes with altered gene expression. In our analysis, a total of 22 artificially selected genes exhibited differential gene expression in the pituitary (*P*<0.05; FDR<0.05; Table 2). Interestingly, four genes (*CTTNBP2NL*, *FRS2*, *KANK4*, and *KATNAL1*) were related to reproduction (Figure 2). Compared to that in the Large White pigs, the expression of the *CTTNBP2NL* gene was increased in BMX pigs. *CTTNBP2NL* has been reported to affect conception rates in bovines (Sugimoto et al., 2013). *FRS2* encodes a docking protein bound to FGF receptors, with a knockout study in mice indicating its involvement in epididymal development and sperm maturation (Xu et al., 2013). In the pituitary tissue, we found that the expression level of *FRS2* in BMX pigs was about twice that found in Large White pigs. In addition, *KANK4* was found to be differentially expressed in the pituitary tissues of BMX and Large White pigs. Although few studies have reported on the function of *KANK4*, it is located in a significant pig quantitative trait locus (QTL) for age at puberty (Bidanel et al., 2008). *KATNAL1*, which encodes Katanin Catalytic subunit A1 like 1, has been implicated in both meiosis and spermiogenesis (Smith et al., 2012).

**Table 2:** Overlap between positively selected genes and differentially expressed genes from pituitary tissue in BMX pigs.

Gene	Normalized count fold change (BMX/LW)	*P*-value^*^
*MGAT5B*	0.08	7.57E-05
*BHLHE22*	2.57	0.000 538
*FAM174B*	0.43	0.000 589
*SFT2D2*	0.28	0.001 381
*TRHDE*	2.13	0.005 172
*COL23A1*	0.12	0.006 991
*FRMD4B*	2.45	0.007 354
*CDKN2B*	0.14	0.007 441
*FRS2*	2.09	0.008 626
*ATG4C*	2.16	0.010 881
*PELI2*	1.90	0.012 313
*FEZ2*	2.12	0.018 321
*LGALS12*	0.13	0.020 835
*TYRP1*	0.13	0.025 025
*KCND3*	1.81	0.030 48
*FNDC1*	0.22	0.030 808
*ATL3*	0.46	0.033 021
*KANK4*	0.34	0.036 521
*KATNAL1*	1.99	0.037 825
*CTTNBP2NL*	2.27	0.039 048
*ANO3*	2.50	0.044 986
*DYNC1I2*	1.67	0.047 03

^*^: *P*-values are corrected by Benjamini-Hochberg FDR<0.05.

For each subgraph, red and green boxes represent gene expression in the pituitaries of BMX and Large White pigs, respectively. Upper whisker is maximum value, black horizontal line in box is median, and lower whisker is minimum value.

## DISCUSSION

We conducted a comparative genomic analysis of BMX pigs with domestic pigs and wild boars from East Asia, and revealed selective signatures potentially associated with selection for early maturity and the two-end black phenotype. During domestication, selection is based on those functions and traits that are favored in pig breeds. As described earlier, selective signatures were detected for the specific phenotype of the BMX pig. This study sheds light on the evolution of puberty in pigs and provides important information and candidate genes for pig breeding.

Puberty is an important trait related to economic output, with early maturity resulting in early slow growth. For instance, Western commercial breeds are characterized with late maturity for enlarged body size (Rubin et al., 2012). The present study revealed four loci (*CTTNBP2NL*, *FRS2*, *KANK4*, and *KATNAL1*) showing strong signatures of selection and functional association with puberty. In addition, the four genes exhibited differential expression between early and late maturity in pig breeds in the pituitary. This result indicates that regulatory elements may have considerable effect on BMX pig development.

Coat color is a trait that is usually under strong selection in different breeds. In previous studies, several genes have been shown to be associated with coat color variants, including *MC1R*, *KIT*, and *EDNRB* (Kijas et al., 1998; Wiener & Wilkinson, 2011; Wilkinson et al., 2013). However, we found no selective signature of *MC1R*. The reason for this may be that selection of *MC1R* occurred during early domestication, and most Chinese indigenous pig breeds share the same haplotype (Li et al., 2010). Earlier research has indicated that mutations in *MITF*, *PAX3*, *EDNRB*, and *KIT* together may explain a large proportion of white spotting phenotypes in horses (Hauswirth et al., 2012). In BMX pigs, the two-end black phenotype is a complex trait resulting in the diversification of white areas on the body. We hypothesize that a correlation exists between coat color variants and different combinations of candidate genes.

One limitation of this study was the identification of the DEG direction in RNA-seq analysis. As both BMX and Large White pigs are domesticated, it was difficult to identify the direction of gene expression without the use of an outgroup. We analyzed the overlap between DEGs (between BMX and Large White pigs) and the artificially selected genes in BMX pigs (compared to LWU and EAW pigs). The shared genes were more likely to be changed in BMX pigs. We did not focus on DEGs that did not exhibit selective signatures in the BMX pig genome. In addition, future analysis of Asian and European pig genome data will help clarify breed development in pigs.

## Supplementary Material

http://www.zoores.ac.cn/fileup/2095-8137/SUPPL/20180803181759.zip
